# Gene expression profiling for the diagnosis of multiple primary malignant tumors

**DOI:** 10.1186/s12935-021-01748-8

**Published:** 2021-01-12

**Authors:** Yu Zheng, Yifeng Sun, Yue Kuai, Guoxiang Fu, Huimin An, Jinyun Chen, Jinying Chen, Jiajun Zhu, Yixin Wo, Yiwang Wu, Kaibin Song, Qinghua Xu, Di Wu, Deshuang Huang, Qifeng Wang, Hongming Pan

**Affiliations:** 1 Department of Medical Oncology, Sir Run Run Shaw Hospital, College of Medicine, Zhejiang University, East Qinchun Road 3, Hangzhou, 310016 Zhejiang China; 2 Canhelp Genomics Research Center, Xinyan Road 22, Hangzhou, 311100 Zhejiang China; 3 Department of Pathology, Sir Run Run Shaw Hospital, College of Medicine, Zhejiang University, Hangzhou, Zhejiang China; 4 Department of General Surgery, Sir Run Run Shaw Hospital, College of Medicine, Zhejiang University, Hangzhou, Zhejiang China; 5 Institute of Machine Learning and Systems Biology, College of Electronics and Information Engineering, Tongji University, Shanghai, China; 6 Department of Pathology, Fudan University Shanghai Cancer Center, Dong’An Road 270, Shanghai, 200032 China; 7 Department of Oncology, Shanghai Medical College, Fudan University, Shanghai, China; 8 Laboratory of Cancer Biology, Institute of Clinical Science, Sir Run Run Shaw Hospital, College of Medicine, Zhejiang University, Hangzhou, Zhejiang China

**Keywords:** Multiple primary malignant tumors, Tissue of origin, Gene expression profiling, 90-gene expression assay, qRT-PCR

## Abstract

**Background:**

The incidence of multiple primary malignant tumors (MPMTs) is rising due to the development of screening technologies, significant treatment advances and increased aging of the population. For patients with a prior cancer history, identifying the tumor origin of the second malignant lesion has important prognostic and therapeutic implications and still represents a difficult problem in clinical practice.

**Methods:**

In this study, we evaluated the performance of a 90-gene expression assay and explored its potential diagnostic utility for MPMTs across a broad spectrum of tumor types. Thirty-five MPMT patients from Sir Run Run Shaw Hospital, College of Medicine, Zhejiang University and Fudan University Shanghai Cancer Center were enrolled; 73 MPMT specimens met all quality control criteria and were analyzed by the 90-gene expression assay.

**Results:**

For each clinical specimen, the tumor type predicted by the 90-gene expression assay was compared with its pathological diagnosis, with an overall accuracy of 93.2% (68 of 73, 95% confidence interval 0.84–0.97). For histopathological subgroup analysis, the 90-gene expression assay achieved an overall accuracy of 95.0% (38 of 40; 95% CI 0.82–0.99) for well-moderately differentiated tumors and 92.0% (23 of 25; 95% CI 0.82–0.99) for poorly or undifferentiated tumors, with no statistically significant difference (p-value > 0.5). For squamous cell carcinoma specimens, the overall accuracy of gene expression assay also reached 87.5% (7 of 8; 95% CI 0.47–0.99) for identifying the tumor origins.

**Conclusions:**

The 90-gene expression assay provides flexibility and accuracy in identifying the tumor origin of MPMTs. Future incorporation of the 90-gene expression assay in pathological diagnosis will assist oncologists in applying precise treatments, leading to improved care and outcomes for MPMT patients.

## Introduction

Multiple primary malignant tumors (MPMTs) are defined as two or more histologically distinct malignancies in one individual. With the development of screening technologies as well as significant treatment advances, early detection and precise treatment have led to a dramatic increase in the population of cancer survivors. In addition to this increase in the population of cancer survivors, the incidence of MPMTs is rising due to increased aging of the population [1]. Worldwide, a meta-analysis of 12 studies revealed that the frequency of MPMTs in a cancer population varies between 2.4 and 8% and is up to 17% within 20 years of follow-up [2]. In China, two epidemiological studies reported that 0.99 to 1.09% of cancer patients could develop a second primary malignancy [3, 4]. The risk of developing a second primary tumor varies across first tumor type; bladder cancer is most common as the first primary tumor, and lung cancer is the most common second primary tumor [5]. Certain patient populations, including male patients and patients with a history of smoking or alcoholism, are also at higher risk of developing MPMTs [2, 6].

When a patient with a prior cancer history has a second malignant lesion, identifying the tumor origin of the new lesion has important prognostic and therapeutic implications and still represents a difficult problem in clinical practice. If the second lesion is a primary cancer, it could be cured by radical operation supplemented by chemotherapy and/or radiotherapy, which is similar to the treatment of a single primary cancer. In contrast, recurrent or metastatic tumors indicate that the primary tumor has progressed to advanced stages. Palliative treatment is the first choice for recurrence or metastasis of the primary tumor. In the clinic, histopathologic analysis can help characterize the tumor origin in most cases. However, tumor heterogeneity and interobserver variation between pathologists can cause confusion, especially when metastatic foci are poorly differentiated or undifferentiated [7].

In recent years, gene expression profiling has been widely studied and has become a powerful tool in distinguishing the origin of tumors. Previous studies have suggested the clinical utility of gene expression profiling in distinguishing synchronous primary malignancies of the ovary and endometrium or metastatic spread from either the ovary or the endometrium, as well as in distinguishing between second primary lung cancer and lung metastasis from head and neck tumors [8, 9]. Nevertheless, few data support the broad application of gene expression profiling for MPMTs.

Recently, Ye et al. established a pan-cancer transcriptome database comprising 5434 specimens representing 21 tumor types (as shown in Additional file 1: Table S1), and adopted the SVM-RFE algorithm (Support Vector Machine Recursive Feature Elimination) to select the Top-10 most predictive genes for each of the 21 tumor types [10]. After removing redundant genes, a list of 90 genes specific to 21 tumor types was identified. The details of 90-gene list were provided in Additional file 2: Table S2. For instance, gene *ACPP* was significantly over-expressed in prostate cancer, while gene *GATA3* was shown to be highly expressed in breast cancer, and gene *SLC3A1* was significantly over-expressed in kidney cancer (Additional file 3: Figure S1). Gene Ontology and KEGG pathway analysis reveal that a diverse group of gene families is represented in the 90-gene list [10]. The most significantly enriched gene categories are those involved in specific biological processes, including tyrosine metabolism, fat digestion and absorption, cytokine-cytokine receptor interaction, extracellular matrix-receptor interaction, and gastric acid secretion. Of interest, but not surprisingly, genes described in oncogenic pathways such as those of bladder cancer, melanoma, and prostate cancer were also significantly over-represented, reflecting their differential involvement in a range of tumor classes. Next, a 90-gene expression assay was established for the classification of 21 common tumor types using quantitative real-time polymerase chain reaction (qRT-PCR) methods with total RNA extracted from formalin-fixed, paraffin-embedded (FFPE) tissue [10]. In a validation study that included 609 clinical samples, the 90-gene expression assay demonstrated an overall accuracy of 90.2% for primary tumors (292/323) and 87.3% for metastatic tumors (255/286). In addition, Wang et al. applied the 90-gene expression assay for the differential diagnosis of metastatic triple-negative breast cancer (TNBC) [11]. The gene expression assay correctly classified 97.6% of TNBC lymph node metastases (41/42) and 96.8% of distant metastatic tumors (30/31). Zheng et al. investigated the 90-gene expression assay for diagnosing the tumor origin of brain tumors [12]. The molecular assay showed 100% accuracy for discriminating primary brain tumors from brain metastases, and correctly predicted primary sites for 89% of brain metastases (39/44). More recently, Ning et al. demonstrated the strengths of the 90-gene expression assay in distinguishing multiple primary squamous cell carcinomas in head and neck, esophageal, and lung cancers [13]. In current study, we aim to evaluate the performance of the 90-gene expression assay and explore its potential diagnostic utility for MPMTs. Our results show that this PCR-based gene expression assay might serve as a useful tool for identifying the origin of MPMTs.

## Methods and materials

### Sample selection

This study was approved by the Institutional Review Board of Sir Run Run Shaw Hospital, College of Medicine, Zhejiang University (Hangzhou, China) and Fudan University Shanghai Cancer Center (Shanghai, China). FFPE tumor samples from 41 patients (84 specimens) archived from July 2013 to July 2020 were used in this study. All samples were excisional biopsies and histopathologically confirmed as MPMTs according to the international diagnostic criteria of Warren and Gate [14]. The tumor that was first diagnosed and associated with the cause of the patient’s initial visit was defined as the first primary cancer, the second diagnosed tumor was considered the second cancer and so forth. According to the Surveillance Epidemiology and End Results (SEER) definitions, all of the first tumors in MPMT patients diagnosed within 6 months were classified as synchronous MPMTs, and tumors diagnosed after more than 6 months were deemed metachronous MPMTs [2]. Hematoxylin and eosin (H&E)-stained slides from tumor samples were reviewed by two senior pathologists to evaluate the percentage of tumor cells and necrotic areas. If fewer than 60% of the tumor cells or greater than 40% of the necrotic area was present on inspection, regions of interest were circled on the H&E-stained slides, and the corresponding areas from unstained FFPE tissue sections were then manually macro-dissected for tumor enrichment.

### Sample preparation and RNA isolation

The tumor tissue from five to fifteen 5-μm-thick paraffin sections was scraped and placed into a 1.5 mL microcentrifuge tube. Total RNA was isolated from FFPE samples using an FFPE Total RNA Isolation Kit (Canhelp Genomics, Hangzhou, China) as described previously [15]. Briefly, FFPE tissue was deparaffinized by sequential washing in xylene at 50 °C for 3 min and twice in 100% ethanol. Proteins were digested with proteinase K solution at 56 °C for 15 min and then for another 15 min at 80 °C followed by treatment with DNase. Total RNA was eluted from the spin column with 40 μL RNase-free water. The total RNA concentration was assessed by a NanoDrop 2000 Spectrophotometer (Thermo Fisher Scientific, Wilmington, DE, United States) at 260 nm, and the purity of extracted total RNA was determined by the ratio of absorbance at 260 nm to that at 280 nm (A260/A280). qRT-PCR analyses were only performed on RNA samples with A260/A280 ratios between 1.7 and 2.1.

### Expression profiling of 90 tumor-specific genes

The qRT-PCR method was used to measure the gene expression levels of 90 tumor-specific genes corresponding to 21 major tumor types as described previously [11]. For each sample, reverse transcription was performed on isolated total RNA using a High-Capacity cDNA Reverse Transcription Kit with RNase Inhibitor (Applied Biosystems, Foster City, CA, United States). Subsequently, qRT-PCR was performed using a 7500 Real-Time PCR system (Applied Biosystems) to measure the expression levels of 90 genes. The PCR cycling conditions were 10 min at 95 °C and 40 cycles of 15 s at 95 °C and 1 min at 60 °C.

### Data analysis

Gene expression data analysis was performed using the R software (version 3.6.0) and packages from the Bioconductor project (version 3.9) [16–18]. The gene expression pattern for each sample was compared with the indicated 21 tumor types by the 90-gene expression signature. Then, similarity scores for each of the 21 tumor types were calculated, which showed similarities in the gene expression pattern between the sample and the indicated tumor type [19]. The similarity score values ranged from 0 (very low similarity) to 100 (very high similarity), which summed up to 100 across all 21 tumor types. The tumor type with the highest similarity score was considered to indicate the tissue of origin. An example of the result from the 90-gene expression assay is shown in Additional file 4: Figure S2.

For each clinical specimen, its pathological diagnosis was considered as the “gold-standard”, and the predicted tumor type by the 90-gene expression assay was compared with its pathological diagnosis. For the entire cohort, the overall accuracy of 90-gene expression assay was defined as the number of matched cases between gene expression assay prediction and pathological diagnosis divided by the total number of estimated cases. More specifically, for a certain cancer type C1, four test statistics were assessed as follows: true positives (TPs; samples belong to C1, and predicted results were C1), true negatives (TNs; samples belong to other cancer types, and predicted results were not C1), false positives (FPs; samples belong to other cancer types, and predicted results were C1), and false negatives (FNs; samples belong to C1, and predicted results were not C1). For each tumor type, sensitivity and specificity can be calculated by applying the following formulas:

$${\text{Sensitivity}}\, = \,{\text{TP}}/\left( {{\text{TP}}\, + \,{\text{FN}}} \right){\text{ and specificity}}\, = \,{\text{TN}}/\left( {{\text{TN}}\, + \,{\text{FP}}} \right).$$

## Results

### Patients and samples

**Fig. 1:**
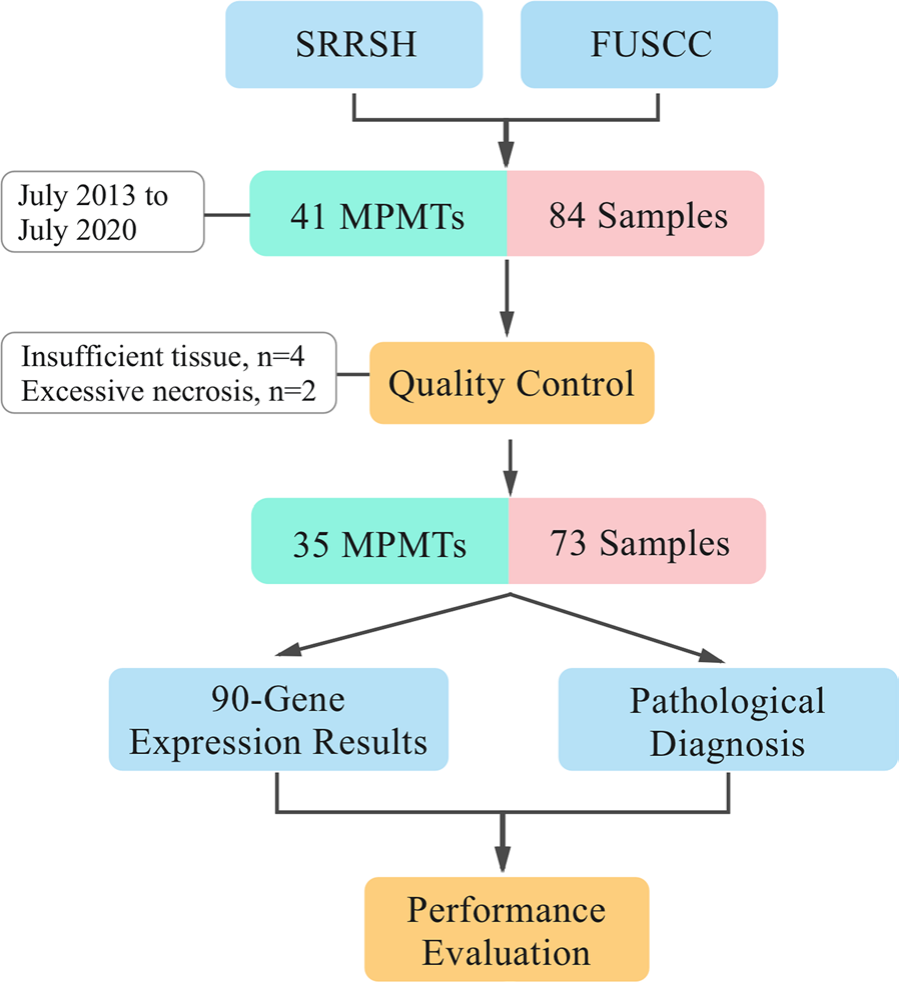
Study design and workflow

**Fig. 2:**
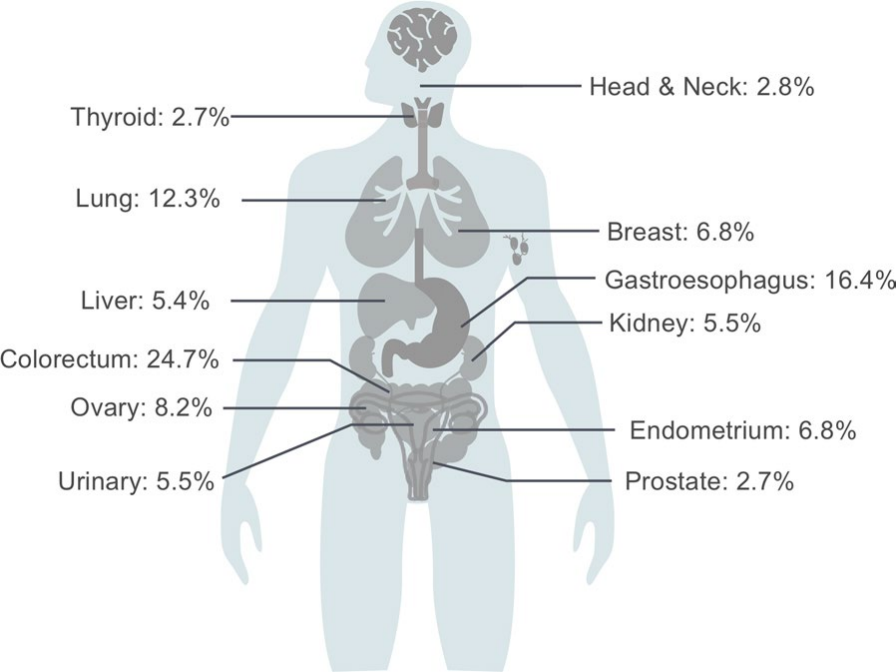
The distribution of tumor origins from 35 MPMT patients

Forty-one patients with a total of 84 specimens were enrolled from Sir Run Run Shaw Hospital, College of Medicine, Zhejiang University and Fudan University Shanghai Cancer Center in this study. As shown in Fig. 1, four specimens were excluded because of insufficient tumor content, and two specimens were excluded due to high content of necrotic tissue in the samples; thus, six corresponding patients were excluded. Thirty-five MPMTs from 73 specimens met all quality control criteria and were analyzed by the 90-gene expression assay. Based on the invasion site of the tumor, 73 specimens were sorted into 12 types, including tumors in the colorectum, gastroesophagus, lung, ovary, endometrium, breast, liver, kidney, urinary, prostate, head & neck and thyroid. Table 1 presents the demographics of the 35 MPMT patients. Among these patients, 30 harbored synchronous MPMTs, and five harbored metachronous MPMTs. Twenty-one patients were male, and fourteen patients were female. The median age at diagnosis was 62.5 (range 33–77) for the first cancer, 63 (range 33–77) for the second cancer and 68 (range 53–70) for the third cancer. The most common invasion sites of the first, second and third tumors were the colorectum, gastroesophagus and colorectum, respectively. Of the 73 specimens, tumors were most frequently located in the colorectum (24.7%, 18 of 73), gastroesophagus (16.4%, 12 of 73) and lung (12.3%, 9 of 73). The distribution of tumor locations is shown in Fig. 2. The most common stages of the first, second and third cancers were II, I and II, respectively. Thirty-two of 35 patients (91.4%) underwent surgery, and 62.9% (22/35) underwent chemotherapy.

**Table 1:** Patients and tumors characteristics included in this study

	First cancer^a^	Second cancer		Third cancer
Gender (%)				
Male		21 (60)		
Female		14 (40)		
Age at diagnosis				
Mean	62.5	63		68
Range	33–77	33–77		53–70
Type (%)				
Synchronous		30 (86)		
Metachronous		5 (14)		
Stage (%)				
I	9 (26)	18 (51)		1 (25)
II	17 (48)	8 (23)		2 (50)
III	6 (17)	6 (17)		1 (25)
IV	3 (9)	3 (9)		0 (0)
Invasion site (%)				
Digestive system	19 (54)	13 (37)		3 (75)
Reproductive	5 (14)	6 (17)		0 (0)
Urinary system	4 (11)	5 (14)		1 (25)
Breast	3 (9)	2 (6)		0 (0)
Lung	3 (9)	6 (17)		0 (0)
Head & neck	1 (3)	3 (9)		0 (0)
Treatment (%)				
Surgery		32 (91)		
Chemotherapy		22 (63)		
Histology (%)				
Well-differentiated		40 (55)		
Poorly differentiated		25 (34)		
Squamous cell carcinoma		8 (11)		

^a^One triple metachronous MPMT patient lacked the first primary cancer specimen

### Performance of the 90-gene expression assay in MPMTs

**Fig. 3:**
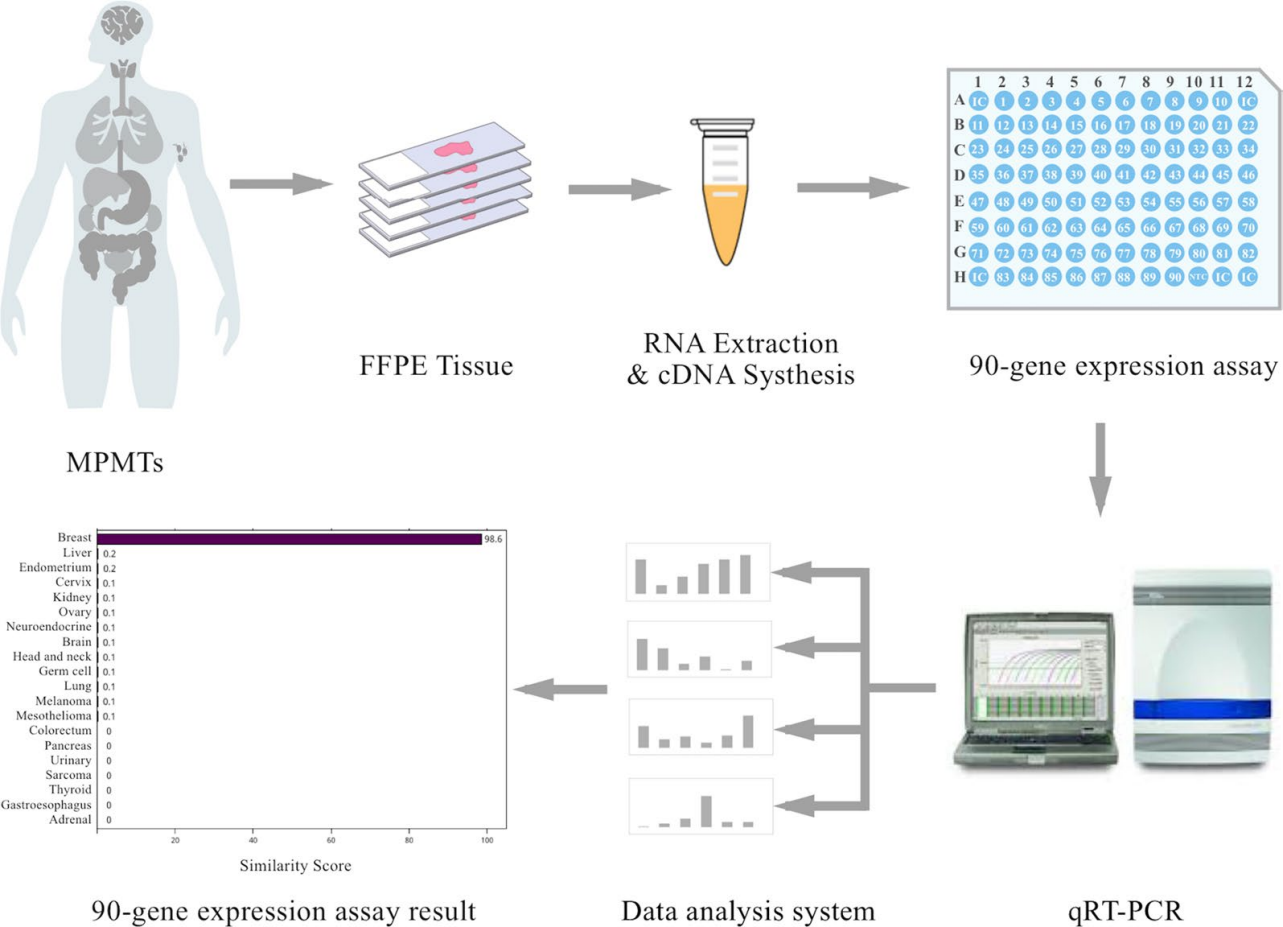
Analysis workflow of the 90-gene expression assay. FFPE tissues collected from MPMT patients were used for RNA extraction and qRT-PCR analysis. The gene expression profiling was analyzed by the 90-gene signature with one similarity score for each of the 21 tumor types. The top three predictions were breast (98.6), liver (0.2) and endometrium (0.2); therefore, the most likely site is breast (98.6)

The overall workflow for the 90-gene expression assay is shown in Fig. 3. The concentrations of total RNA from 73 samples ranged from 3.3 to 255.5 ng/μL, with a median of 66.6 ng/μL. The median A260/A280 ratio (purity of RNA) was 1.98 (range 1.74–2.04).

With the 90-gene expression assay, 20 specimens were classified as colorectal tumors, 11 as gastroesophageal tumors, 7 as lung tumors, 6 as breast tumors, 6 as ovary tumors, 5 as endometrium tumors, 4 as urinary tumors, 4 as kidney tumors, 3 as liver tumors, 3 as head & neck tumors, 2 as prostate tumors and 2 as thyroid tumors. For the entire cohort, the 90-gene expression assay showed an overall accuracy of 93.2% [68 of 73, 95% confidence interval (CI) 0.84–0.97]. As shown in Table 2, the sensitivity of 90-gene expression assay were 100% for classifying tumors from the colorectum, ovary, endometrium, breast, kidney, urinary tract, prostate, head & neck and thyroid. Furthermore, the 90-gene expression assay correctly classified 83.3% of the gastroesophageal cancer cases, 77.8% of the lung cancer cases and 75.0% of the liver cancer cases. As shown in Table 3, 5 out of 73 specimens had discordant molecular classifications compared with the pathological diagnosis, including two lung tumors, two gastroesophageal tumors and one gallbladder tumor.

**Table 2:** Performance of the 90-gene expression assay in MPMTs

Tumor type	Number	# corrected predicted	Sensitivity (%)
Colorectum	18	18	100.0
Gastroesophagus	12	10	83.3
Lung	9	7	77.8
Ovary	6	6	100.0
Endometrium	5	5	100.0
Breast	5	5	100.0
Liver	4	3	75.0
Kidney	4	4	100.0
Urinary	4	4	100.0
Prostate	2	2	100.0
Head & neck	2	2	100.0
Thyroid	2	2	100.0
Total accuracy	73	68	93.2

**Table 3:** Investigation of cases with discordant 90-gene expression assay results

ID	Gender	Type	First/second/third	Age	Pathological diagnosis	Grade	Gene expression assay results	Stage	Treatment
1	Female	Metachronous	Second cancer	67	Left main bronchus mucoepidermoid carcinoma	Highly and moderately differentiated	Breast	I	Surgery & chemotherapy
2	Male	Synchronous	Second Cancer	68	Gastric antrum adenocarcinoma	Poorly differentiated	Colorectum	I	Surgery & chemotherapy
3	Female	Synchronous	Primary cancer	50	Gallbladder adenocarcinoma	Poorly differentiated	Gastroesophagus	II	Surgery
4	Female	Synchronous	Second cancer	72	Lung adenocarcinoma	Highly and moderately differentiated	Colorectum	I	Surgery & chemotherapy
5	Male	Metachronous	Primary Cancer	58	Esophageal squamous cell carcinoma	Squamous cell carcinoma	Head & neck	II	Surgery & chemotherapy

The performance of 90-gene expression assay stratified by histopathological features was further investigated. Among 73 specimens, 40 (55%) were well-moderately differentiated tumors, 25 (34%) were poorly or undifferentiated tumors, and 8 (11%) were squamous cell carcinoma. Regarding to the predictions of 90-gene expression assay, the overall accuracy was 95.0% (38 of 40; 95% CI 0.82–0.99) for well-moderately differentiated tumors and 92.0% (23 of 25; 95% CI 0.82–0.99) for poorly or undifferentiated tumors, with no statistically significant difference (p-value > 0.5). In addition, the overall accuracy was 87.5% (7 of 8; 95% CI 0.47–0.99) for squamous cell carcinoma.

### Specific case

**Fig. 4:**
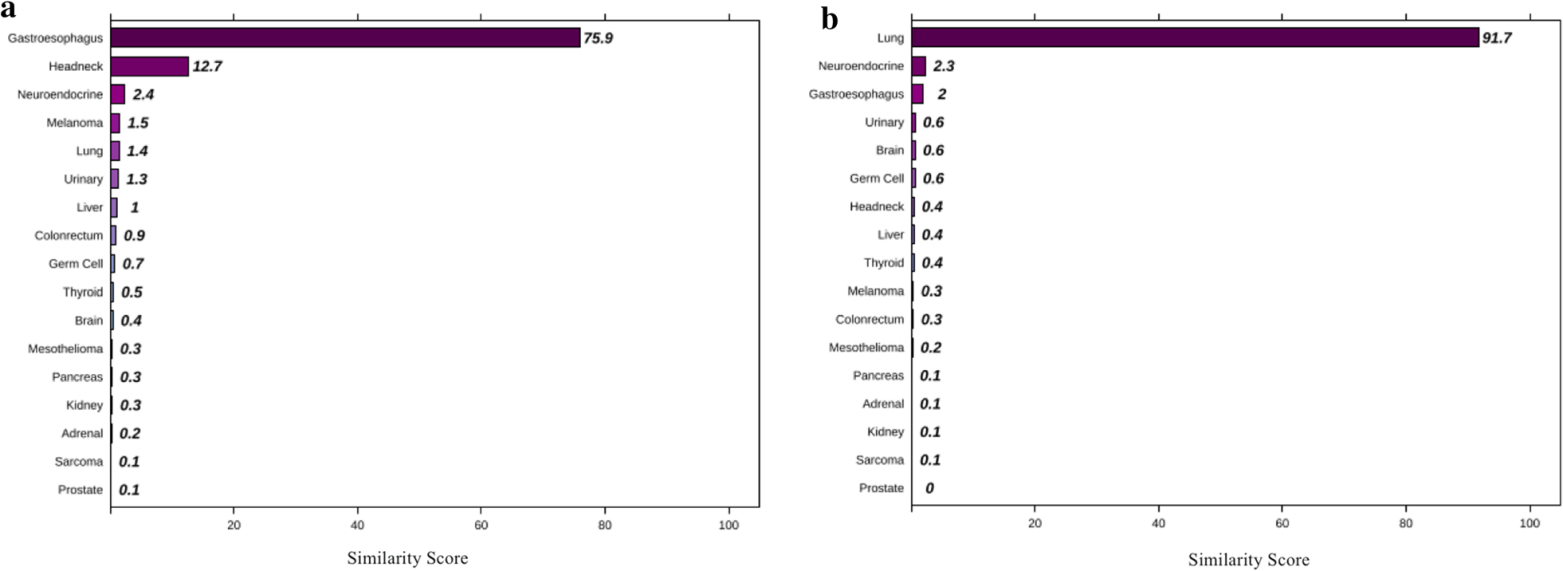
The 90-gene expression assay results of specific cases. **a** Esophagus lesion and **b** lung lesion

A 77-year-old man noticed chest tightness, shortness of breath, nausea/vomiting and fever. He underwent endoscopic biopsies in the gastroesophageal region (29–35 cm from the incisors) and was diagnosed with squamous cell carcinoma (SCC). In the meantime, a chest computed tomography (CT) scan found a lesion in the left upper lung, and the lung biopsy was diagnosed as SCC. Through comprehensive clinical and pathology examinations, the clinician confirmed that the patient had multiple synchronous primary tumors according to the Warren and Gates criteria [14]. FFPE tumor tissues taken from the esophagus and lung were analyzed by the 90-gene expression assay, and the predictions showed that the two specimens were gastroesophageal cancer and lung cancer (Fig. 4).

SCC comprises a wide range of tumors originating from diverse anatomical locations that share a common histomorphology and expression of squamous cell differentiation markers, making it difficult to distinguish whether the subsequent SCC is a primary tumor or metastatic lesion. This patient had two simultaneous lesions on the esophagus and lung. Histopathologically, esophageal SCC often metastasizes to the lung. Pathological diagnosis could only confirm the two lesions in the esophagus and lung as SCCs. It was challenge to determine whether the two lesions were synchronous SCCs or represented SCC metastasis based on immunohistochemistry (IHC) and morphology assessment. The clinical outcome of synchronous esophageal and lung SCCs is better than that of metastatic cancer, and the diagnosis of the tumor will directly affect the treatment options. If the new lesion is a second primary tumor, surgical resection supplemented by chemotherapy or radiotherapy has been the preferred therapeutic regimen instead of palliative treatment. Thus, for patients highly suspected of having metastatic cancer, the 90-gene expression assay can be useful to identify the tissue of origin more quickly when imaging and IHC examinations are ineffective, and support the choice of precise treatment.

## Discussion

Currently, with the significant development in tumor screening and diagnostic tools, the detection rate of MPMTs is higher than before. Additionally, the appearance of new treatment methods, including targeted therapy and immunotherapy, has helped cancer patients achieve longer survival. Therefore, the likelihood of cancer survivors having a second malignancy is higher than before because of the longer follow-up times. Lindsay et al. found that the occurrence of cancer survivors developing second cancers rose dramatically from 9% in 1975–1979 to 19% in 2005–2009 [1]. Identifying the tumor origin of the new lesion for patients with cancer history is crucial for selecting the treatment strategy. A second primary cancer could be cured by radical operation supplemented by chemotherapy and/or radiotherapy, which is similar to the treatment of a single primary cancer. In contrast, recurrent or metastatic tumors indicate that the primary tumor has progressed to advanced stages. Palliative treatment is the first choice for recurrence or metastasis of the primary tumor. For people who develop a second primary cancer, 13% die from their original cancer, and 55% die from their second primary cancer [5]. Thus, how to accurately diagnose tumors as metastatic or a second primary cancer in a timely manner is becoming increasingly important.

Histopathologic examinations, such as morphological and IHC analyses, are the cornerstone of traditional cancer diagnosis. However, a meta-analysis showed that IHC provides correct tissue identification in 65.6% of metastatic cancers [20], and recent studies suggest that molecular profiling outperforms classification by IHC, in particular in cases with poorly differentiated tumors [7, 21]. In addition, for patients with multiple SCC lesions, morphological and IHC analyses cannot provide a definite diagnosis of primary site given similar histologic appearances among across several types of SCCs.

Previous studies have demonstrated that gene expression profiling between primary and metastatic tumors shows a high degree of similarity [7, 10]. Efforts have been made to use gene expression profiling to distinguish the second primary tumor from metastatic recurrence. Charles et al. found that gene expression profiling could accurately identify 89% of metastatic tumors, whereas IHC achieved 83% agreement. In poorly differentiated or undifferentiated subsets, gene expression profiling correctly distinguished 91% of cases compared to 71% using the IHC method [21]. Anita et al. developed a gene expression profiling test to distinguish between synchronous primary malignancies of the ovary and endometrium or metastatic spread from either the ovary or the endometrium and achieved an accuracy of 94.7% (71/75) [8]. In addition, Anital et al. reported a new approach to determine if a lung nodule was a second primary or a lung metastasis from head & neck tumors with an overall accuracy of 82.9% (63/76) [9]. Regardless, few studies have validated the performance of gene expression profiling in MPMTs across a broad spectrum of tumor types.

Here, we describe the investigation of an effective approach for the molecular classification of MPMTs. Similar to findings from previous studies [11, 12], the success rate of the 90-gene expression assay was excellent (87%), even for samples archived five years prior to analysis, indicating high reliability of the assay with FFPE samples. This may be critical for widespread access and application in clinical practice. The 90-gene expression assay classified 73 MPMT samples into twelve tumor types and reached an overall accuracy of 93.2% (68/73) when compared with the pathological diagnosis. To the best of our knowledge, this is the first report of a gene expression assay that can be used to classify MPMTs into a wide range of cancer types. Our findings suggest that the 90-gene expression signature may serve as a useful tool for identifying the tumor origin of MPMTs.

Our results represent an encouraging primary step, but this study still has several limitations. First, MPMT patients were enrolled from two hospitals. The number of several tumor types was relatively small to obtain a solid conclusion. In the future, additional multi-center validations should be performed to further evaluate the performance of the 90-gene expression assay. Second, in current study, RNAs extracted from long-term archived FFPE samples are likely degraded, and thus might diminish the distinctness of gene expression pattern cross different cancer types. For routine diagnostic evaluation, fresh tumor or newly prepared FFPE sample reserving high quality RNAs are highly recommended for precise gene expression analysis.

## Conclusion

In conclusion, the results of this study demonstrate the promising performance of the 90-gene expression assay in identifying the tumor origin of MPMTs. In cases which the morphological and IHC work-up cannot clearly confirm the tumor origin, the 90-gene expression assay may serve as a useful tool for identifying primary site of new lesions for cancer patients, especially for SCC patients. Future incorporation of the 90-gene expression assay in tumor origin diagnosis will assist oncologists in applying precise treatments, leading to improved care and outcomes for MPMT patients.

## Supplementary Information

**Additional file 1: Table S1.** List of 21 tumor types.

**Additional file 2: Table S2.** Details of 90-gene list.

**Additional file 3: Figure S1.** Examples of genes that differentially expressed across multiple tumor types. Gene *ACPP* was significantly over-expressed in prostate cancer, gene *GATA3* was shown to be highly expressed in breast cancer, and gene SLC3A1 was significantly over-expressed in kidney cancer

**Additional file 4: Figure S2.** Example of the 90-gene expression assay result. The gene expression pattern was analyzed with the 90-gene signature, with one similarity score for each of the 21 tumor types. The top five tumor origins with highest similarity scores are as follows: Breast (90.3), Cervix (1.7), Ovary (1.6), Germ cell (1.1), and Neuroendocrine (1), thus indicating that the most likely tissue of origin is breast (90.3).

## Data Availability

Not applicable.

## Abbreviations

CT: Computed tomography
FFPE: Formalin-fixed, paraffin-embedded
H&E: Hematoxylin and eosin
IHC: Immunohistochemistry
MPMTs: Multiple primary malignant tumors
qRT-PCR: Quantitative real-time polymerase chain reaction
SCC: Squamous cell carcinoma
SEER: Surveillance Epidemiology and End Results
SVM-RFE: Support Vector Machine Recursive Feature Elimination
TNBC: Triple-negative breast cancer

## References

[1] Morton LM, Onel K, Curtis RE, Hungate EA, Armstrong GT (2014). The rising incidence of second cancers: patterns of occurrence and identification of risk factors for children and adults. Am Soc Clin Oncol Educ Book.

[2] Vogt A, Schmid S, Heinimann K, Frick H, Herrmann C, Cerny T, Omlin A (2017). Multiple primary tumours: challenges and approaches, a review. ESMO Open.

[3] Liu Z, Liu C, Guo W, Li S, Bai O (2015). Clinical analysis of 152 cases of multiple primary malignant tumors in 15,398 patients with malignant tumors. PLoS ONE.

[4] Zhai C, Cai Y, Lou F, Liu Z, Xie J, Zhou X, Wang Z, Fang Y, Pan H, Han W (2018). Multiple primary malignant tumors - a clinical analysis of 15,321 patients with malignancies at a Single Center in China. J Cancer.

[5] Donin N, Filson C, Drakaki A, Tan HJ, Castillo A, Kwan L, Litwin M, Chamie K (2016). Risk of second primary malignancies among cancer survivors in the United States, 1992 through 2008. Cancer.

[6] Irelli A, Sirufo MM, D’Ugo C, Ginaldi L, De Martinis M (2020). Sex and gender influences on cancer immunotherapy response. Biomedicines.

[7] Weiss LM, Chu P, Schroeder BE, Singh V, Zhang Y, Erlander MG, Schnabel CA (2013). Blinded comparator study of immunohistochemical analysis versus a 92-gene cancer classifier in the diagnosis of the primary site in metastatic tumors. J Mol Diagn.

[8] Lal A, Panos R, Marjanovic M, Walker M, Fuentes E, Kapp DS, Henner WD, Buturovic LJ, Halks-Miller M (2012). A Gene expression profile test for the differential diagnosis of ovarian versus endometrial cancers. Oncotarget.

[9] Lal A, Panos R, Marjanovic M, Walker M, Fuentes E, Kubicek GJ, Henner WD, Buturovic LJ, Halks-Miller M (2013). A gene expression profile test to resolve head & neck squamous versus lung squamous cancers. Diagn Pathol.

[10] Ye Q, Wang Q, Qi P, Chen J, Ren W, Symposium MXW, 2018. Development and validation of a 90-gene real-time PCR assay for tumor origin identification.

[11] Wang Q, Xu M, Sun Y, Chen J, Chen C, Qian C, Chen Y, Cao L, Xu Q, Du X, Yang W (2019). Gene expression profiling for diagnosis of triple-negative breast cancer: a multicenter retrospective cohort study. Front Oncol.

[12] Zheng Y, Ding Y, Wang Q, Sun Y, Teng X, Gao Q, Zhong W, Lou X, Xiao C, Chen C, Xu Q, Xu N (2019). 90-gene signature assay for tissue origin diagnosis of brain metastases. J Transl Med.

[13] Qu N, Huang D, Xu Q, Wang J, et CCAB. Gene expression profiling of cells of origin of squamous cell carcinomas in head-and-neck, esophagus, and lung; 2020. http://www.academic.oup.com. 10.1093/abbs/gmz153 31960064

[14] Warren S (1932). Multiple primary malignant tumors. A survey of the literature and a statistical study. Am J Cancer.

[15] Wang Q, Gan H, Chen C, Sun Y, Chen J, Xu M, Weng W, Cao L, Xu Q, Wang J (2017). Identification and validation of a 44-gene expression signature for the classification of renal cell carcinomas. J Exp Clin Cancer Res.

[16] Ihaka R, Gentleman R (1996). R: a language for data analysis and graphics. arXiv.

[17] Reimers M, Carey VJ (2006). Bioconductor: an open source framework for bioinformatics and computational biology. Meth Enzymol.

[18] Chang C-C, Lin C-J (2011). LIBSVM: a library for support vector machines. ACM Trans Intell Syst Technol TIST.

[19] Xu Q, Chen J, Ni S, Tan C, Xu M, Dong L, Yuan L, Wang Q, Du X (2016). Pan-cancer transcriptome analysis reveals a gene expression signature for the identification of tumor tissue origin. Mod Pathol.

[20] Anderson GG, Weiss LM (2010). Determining tissue of origin for metastatic cancers: meta-analysis and literature review of immunohistochemistry performance. Appl Immunohistochem Mol Morphol.

[21] Handorf CR, Kulkarni A, Grenert JP, Weiss LM, Rogers WM, Kim OS, Monzon FA, Halks-Miller M, Anderson GG, Walker MG, Pillai R, Henner WD (2013). A multicenter study directly comparing the diagnostic accuracy of gene expression profiling and immunohistochemistry for primary site identification in metastatic tumors. Am J Surg Pathol.

