# Supplementary figures and images for: Gene expression profiling for the diagnosis of multiple primary malignant tumors
Source: Cancer Cell Int. 2021 Jan 12;21:47. doi: 10.1186/s12935-021-01748-8 (PMC7846996; doi:10.1186/s12935-021-01748-8)

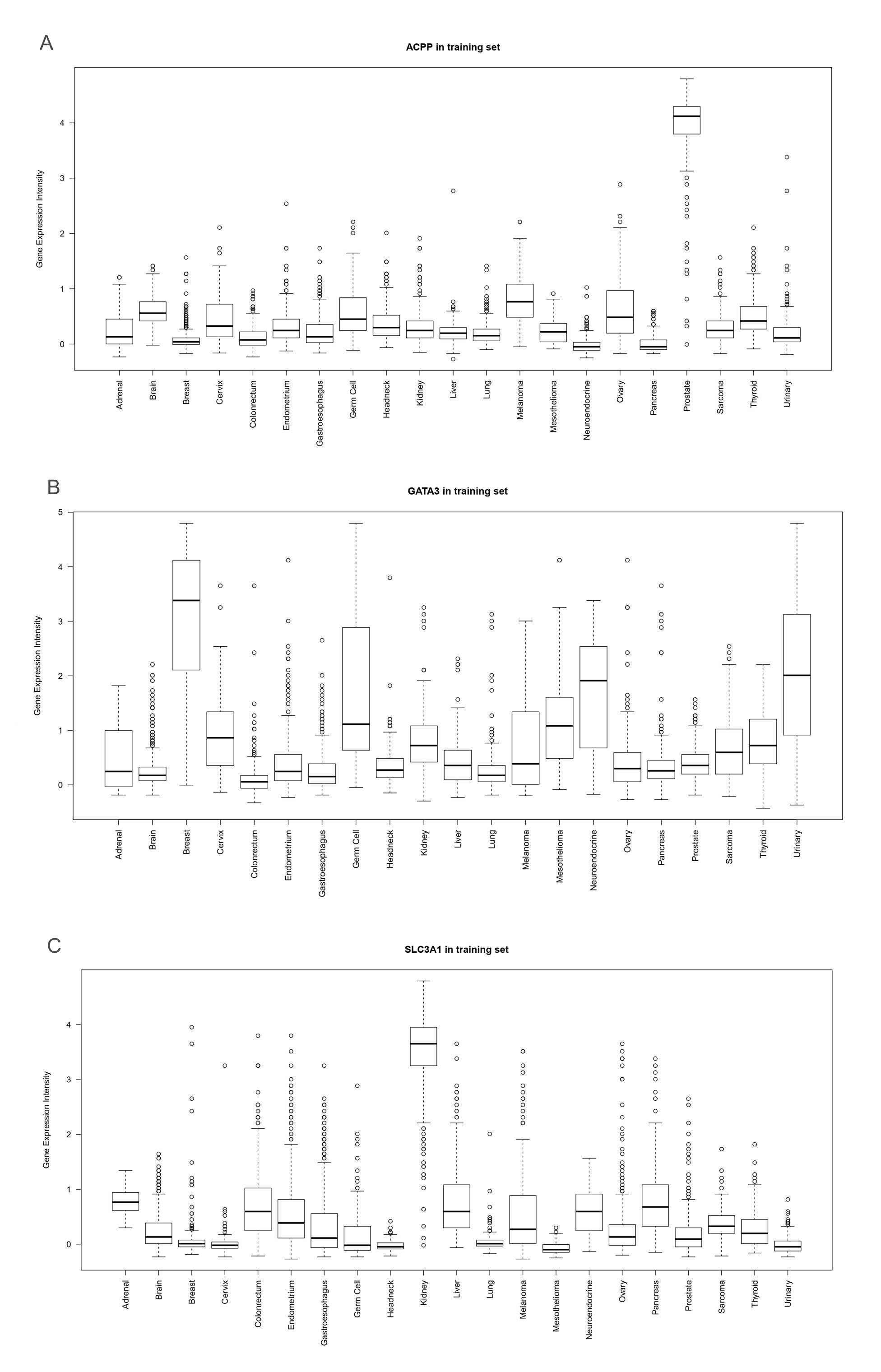

Supplement: Supplementary file 3 — Additional file 3: Figure S1. Examples of genes that differentially expressed across multiple tumor types. Gene ACPP was significantly over-expressed in prostate cancer, gene GATA3 was shown to be highly expressed in breast cancer, and gene SLC3A1 was significantly over-expressed in kidney cancer [file 12935_2021_1748_MOESM3_ESM.jpg]

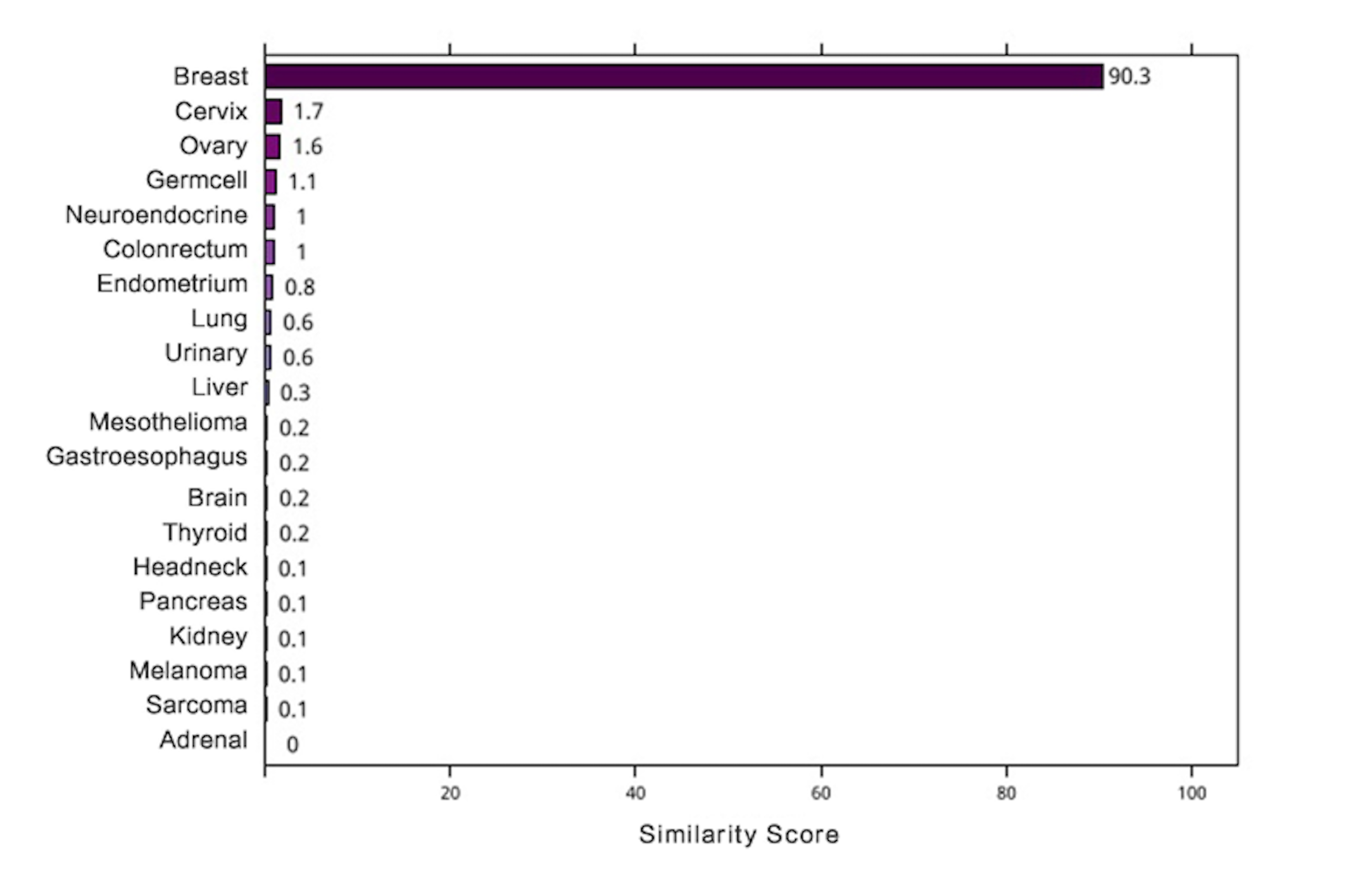

Supplement: Supplementary file 4 — Additional file 4: Figure S2. Example of the 90-gene expression assay result. The gene expression pattern was analyzed with the 90-gene signature, with one similarity score for each of the 21 tumor types. The top five tumor origins with highest similarity scores are as follows: Breast (90.3), Cervix (1.7), Ovary (1.6), Germ cell (1.1), and Neuroendocrine (1), thus indicating that the most likely tissue of origin is breast (90.3). [file 12935_2021_1748_MOESM4_ESM.jpg]
